# Péritonite sur corps étranger intra rectal: à propos d’un cas

**DOI:** 10.4314/pamj.v8i1.71049

**Published:** 2011-01-24

**Authors:** Hanane Bouamama, Aziz Elmadi, Mohamed Rami, Khalid Khatala, Youssef Bouabdallah, Abdrahman Afifi

**Affiliations:** 1 Service de chirurgie pédiatrique, CHU Hassan II de Fès, Maroc

**Keywords:** Corps étranger, rectum, péritonite

## Abstract

Abstract La présence de corps étrangers intra rectaux est peu courante dans les pays en développement et surtout chez l’enfant, les praticiens qui y exercent y sont rarement confrontés. Nous en rapportons un cas observé chez une fille de 10 ans qu’on avait admis en péritonite et chez qui le diagnostic d’un corps étranger intra rectal a été fait en peropératoire. On discutera à partir de cette observation les circonstances de survenu, les moyens diagnostiques et les particularités de prise en charge avec revue de littérature.

## Introduction

Il est habituel de classer les péritonites en fonction du mode de contamination péritonéale. Elles sont en règle, secondaires à une affection d’un viscère intrapéritonéal. Les corps étrangers (CE) intra-abdominaux constituent une cause rare, mais non exceptionnelle, de péritonite. Nous rapportons un cas insolite de péritonite par bout de tuyau non pas pour sa traduction clinique, mais pour sa pathogénie.

## Patient et observation

Il s’agit d’une fille âgée de 10 ans, sans antécédents pathologiques notables, qui présente quatre jours avant son admission une douleur au niveau de la fosse iliaque droite, qui s’est généralisée à tout l’abdomen, associé à des vomissements alimentaires sans troubles de transit, le tout évoluant dans un contexte de fièvre non chiffré, chez qui l’examen clinique a révélé une fièvre à 39°, avec une défense abdominale généralisée.

Un bilan biologique a était fait objectivant une hyperleucocytose à 15380 éléments/mm^3^ avec des polynucléaires neutrophiles à 81%, une anémie hypochrome microcytaire (Hb à 8,6 g/dl VGM à 63 et TCMH à 20,5) et une CRP augmenté à 313, le reste du bilan biologique était sans particularités.

**Figure 1:**
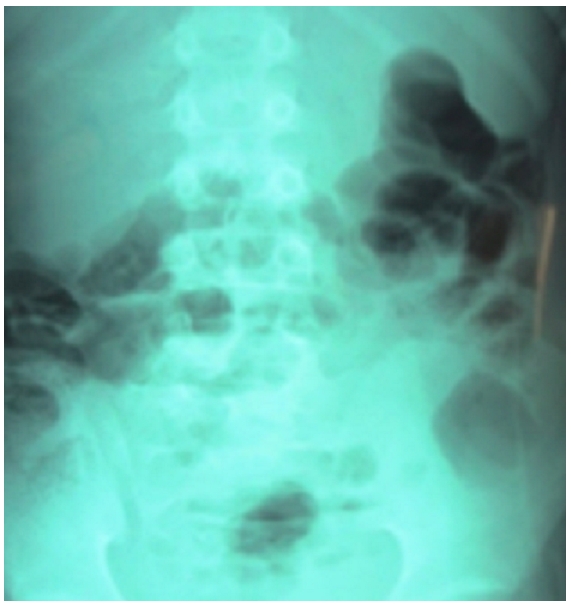
Abdomen sans préparation à l'admission aux urgences (patiente en position debout) montrant une agglutination des anses au niveau de la fosse iliaque droite sans images suspectes d'un corps étranger quelconque

L’abdomen sans préparation a montré quelques niveaux hydro-aériques de type grêliques au niveau de la fosse iliaque droite et au niveau pelvien avec un comblement de la gouttière pariéto-colique droite (figure 1). L’échographie abdominale a décelé un épanchement au niveau des fosses iliaques droite et gauche et du cul de sac de douglas avec un appendice qui mesure 7,4 mm de diamètre.

**Figure 2:**
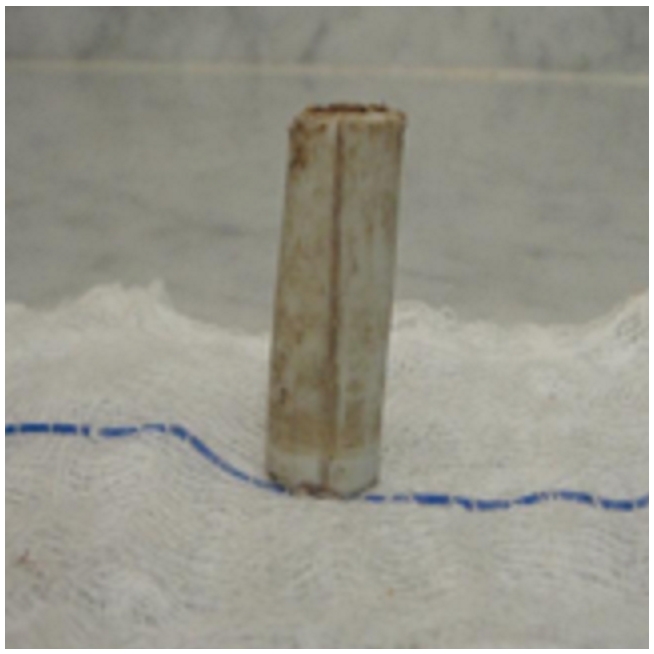
Corps étranger trouvé dans le cul de sac de Douglas, de forme cylindrique mesurant 4cm de longueur sur 1cm de diamètre, de consistance rigide et un aspect de tuyau en caoutchouc

Sur ce le diagnostic d’une péritonite d’origine fort probablement appendiculaire a était retenu. Après une réanimation hydro électrolytique, l’enfant fut admis au bloc opératoire. Après une incision médiane à cheval de l’ombilic et ouverture du péritoine, l’exploration trouve un épanchement purulent au niveau du cul de sac de douglas d’environ 50cc qui émergeait un bout de tuyau en plastique, mesurant 4 cm de longueur sur 1cm de diamètre (figure 2), l’appendice était retro caecale d’allure normale, il y avait une inflammation très importante de l’utérus avec les trompes et les ovaires.

Apres exploration du grêle et tout le cadre colique on a découvert une perforation du rectum très bas situé sur sa face antérieure.

Après avoir ravivé les berges on a pu suturer la perforation et protéger les sutures par une colostomie de décharge, puis on a fermé après un lavage abondant au sérum salé sur deux lames de Delbet. On a conclu que le corps étranger a était introduit par l’anus puis ce dernier avait perforé le rectum pour se trouver dans le cul de sac de douglas.

L’évolution post opératoire était sans particularités, la colostomie a était fermé un mois après avec une bonne évolution clinique, l’enfant fut interrogé à plusieurs reprises pour pouvoir déceler les circonstances de l’accident, notamment pour savoir s’il s’agissait de sévices sexuels mais on a été confronté à chaque fois à la réticence de ce dernier, sur ce on a adressé l’enfant à un médecin psychiatre pour suivi.

## Discussion

Actuellement, l'insertion de CE par l'anus est de plus en plus rencontré surtout chez l’adulte [1]. Des objets peuvent être insérés dans le rectum à des fins diagnostiques, thérapeutiques, sexuelles ou lors de voies de fait criminelles ou encore lors de circonstances accidentelles. Chez l’adulte, les CE enclavés dans le rectum sont pour la plupart insérés à des fins sexuelles et concernent surtout les sujets de sexe masculin [2], par contre chez l’enfant ces CE sont insérés en général par une tiers personne lors d’un abus sexuel.

Les corps étrangers (CE) insérés dans le rectum sont de nature variable dépendant de l’imagination déviante de ces patients : bouteille de whisky, sex-toys, ampoule, magazine, paire de lunettes pour ne citer que cela [3,4]. L'érotisme anal et l'insertion de ces CE peuvent entraîner de graves lésions recto-sigmoïdiennes par perforation survenant chez 3 à 17% des cas [5].

Le pronostic vital peut être ainsi mis en jeu d’où l’intérêt d’une prise en charge rapide [5]. Ainsi, devant un symptôme rectal atypique, il faut faire un interrogatoire poussé avec le patient, suivi d’un examen physique complet sans oublier le toucher rectal qui peut poser le diagnostic quand le corps étranger est bas situé. La radiographie standard va confirmer la présence du corps étranger s’il est radio-opaque et préciser sa nature, sa situation et ses dimensions.

Des signes indirects peuvent être observés en cas de CE radio-transparent (légumes, objets en caoutchouc) [6] ou en cas de complication évoquant un syndrome occlusif ou une péritonite (pneumopéritoine). Pour notre part, l’enfant a été admis en stade de péritonite et la cause qui est la perforation du rectum par corps étranger n’a été découverte qu’en peropératoire.

Si le diagnostic a été établi avant le stade de complication, l’extraction s’imposerait sauf que cette dernière pose aussi une problématique. Elle doit être réalisée par voie basse dans la mesure du possible. Une anesthésie locorégionale ou même générale au bloc opératoire s’impose pour un relâchement des sphincters anaux [6]. La visualisation par le biais d'un recto-sigmoïdoscope rigide peut y être associée en veillant à ne pas pousser le corps étranger en proximal [7].

Des succès d’extraction par endoscopie souple ont été rapportés mais concernent surtout les CE de petite taille [8]. Une variété d’instruments orthopédique et/ou gynécologique peut être utilisée pour avoir d’une part une bonne exposition anale et d’autre part une bonne préhension du corps étranger permettant son extraction par voie endoluminale [9]. Certains facteurs comme la taille, la forme et la migration des corps étranger peuvent rendre difficile la recherche et l’extraction par voie basse. En cas d’échec, une laparotomie s’avère nécessaire [7,10] pour repousser l’objet vers l’ampoule rectale sans ouvrir le colon. Néanmoins, pour les corps étrangers de grande taille, une colostomie peut s’avérer nécessaire. La décision de créer une stomie de dérivation dépend du degré de traumatisme du périnée, de la chronicité de l’acte et de l’apparence du rectum et du colon en peropératoire [11].

Un soutien psychologique et une thérapie sexuelle doivent être suggérés aux patients. L’intéressé refuse souvent cette démarche dans notre contexte socio-culturel riche en tabou et en interdits [12].

## Conclusion

Si les CE colorectaux introduits par voie anale sont des évènements banals dans les pays dits développés, ils restent peu fréquents dans notre contexte. Cette observation en a illustré un cas montrant les difficultés rencontrées dans la prise en charge et dans le suivi de ces patients. Les praticiens exerçant dans nos contrées peuvent donc y être confrontés. Si le diagnostic est relativement facile après un interrogatoire soigneux et un examen clinique rigoureux, une prise en charge rapide permet d’éviter de graves complications.

## Conflits d’intérêt

Les auteurs ne déclarent aucun conflit d’intérêt.

## Contribution des auteurs

Bouamama Hanane est participe à la prise en charge opératoire du patient et a qui a rédigé l'article. Elmadi Aziz a contribué à la prise en charge post opératoire du malade et participé à la rédaction de l'article. Rami mohammed a contribué à la prise en charge post opératoire du malade et à la rédaction de l'article. Khattala khalid a contribué à la prise en charge post opératoire du malade et à la rédaction de l'article. Bouabdallah youssef a contribué à la prise en charge post opératoire du malade et à la rédaction de l'article. Afifi abdrrahman a contribué à la prise en charge opératoire du malade et à la correction de l'article. Tous les auteurs ont lu et approuvé la version finale du manuscrit.
